# Supplementary material for: Are carbon emissions trading and green financial instruments synergistic? -Comprehensive quantitative research based on content analysis
Source: PLoS One. 2024 Mar 7;19(3):e0298601. doi: 10.1371/journal.pone.0298601 (PMC10919601; doi:10.1371/journal.pone.0298601)
Supplement: S1 Data — (ZIP) [file pone.0298601.s001.zip › Data/地级市Z_F工作报告.zip!/─■╧─/╣╠╘¡╩╨/└┤╫╘╣1⁄2╓┌║┼í╛┬φ┐╦╩2╛▌═°í┐_2014.docx]

各位代表：

　　现在，我代表市人民政府向大会报告工作，请予审议，并请政协委员和列席人员提出意见。

　　2013年主要工作

　　一年来，市人民政府认真贯彻落实自治区党委、政府和市委一系列决策部署，自觉接受人大常委会及代表、政协及委员的监督，齐心协力，攻坚克难，较好地完成了三届人大一次会议确定的各项目标任务，全市经济社会呈现出稳中求快、向好的良好局面。

　　实现地区生产总值182.95亿元，增长11.8%;地方公共财政预算收入13.06亿元，增长26.3%;全社会固定资产投资231.2亿元，增长31.9%;城镇居民人均可支配收入18789.1元，增长11.5%;农民人均纯收入5359.2元，增长14.3%;社会消费品零售总额49.53亿元，增长14.5%。上述指标除GDP增速、城镇居民人均可支配收入增速分别未完成14%(主要是建筑业未完成计划，影响GDP增速2.2个百分点)和13%的计划外，其他指标全部实现计划目标。城镇登记失业率3.97%，人口自然增长率9.73‰，居民消费价格涨幅3.8%，万元GDP能耗1.267吨标煤，这四项指标完成自治区控制目标。

　　一、经济发展速度加快，质量提升。以提高经济运行质量和效益为中心，力保增长。一是协调落实有效。成立经济协调领导小组，实行市级领导包抓县(区)、企业和重点工作，对经济运行月分析、季报告，做到年初分解任务、年中督促检查、年底考核落实。地区生产总值、固定资产投资、农业增加值、工业增加值、第三产业增加值、地方公共财政预算收入、城镇居民人均可支配收入和农民人均纯收入等八项指标增幅居全区五市第一。二是项目带动有效。围绕10件大事，开展重点项目建设大会战，落实建设任务，推动项目建设进度。全年开工500万元以上项目497个，其中100个重点项目开工96个，累计完成投资150亿元，创历史新高。三是产业支撑有效。出台加快特色农业、工业经济发展意见，完善旅游产业发展规划。安排5050万元支持发展，初见成效：特色农业规模效应凸现。主抓基地建设和市场营销环节，主打六盘山农产品品牌。肉牛新增4.5万头，达到90.5万头;马铃薯种薯新增3.2万亩，达到28.2万亩;冷凉蔬菜新增3.9万亩，达到52.6万亩;大田中药材新增2.9万亩，达到10万亩。特色农业为农民人均增收2134元，增长13%。培育自治区级龙头企业20家，新建农业科技示范园6个。农业机械化率57.6%，提高3.2个百分点，农产品加工转化率44.3%，提高1.5个百分点。粮食总产7.99亿公斤。完成农业增加值44.72亿元，增长6%。西吉县被列为国家粮食增产模式科技攻关试点县，泾源县黄牛养殖跻身“全国优秀农业标准化示范区”，彭阳县被命名为“中国辣椒之乡”。工业发展量增质升。认真落实首次召开的工业强市会议精神，启动建设轻工产业园，续建盐化工示范区和各县(区)产业园基础设施，进驻企业181家。中铝煤电铝一体化项目经过多次对接争取，专家现场察勘，形成评审报告，报国家发改委待批。金昱元20万吨PVC项目顺利推进。原州中能振发光伏发电、西吉仁初包装、隆德日普电动车和泾源恒辉实业LED节能照明灯具等项目投产，彭阳王洼煤矿600万吨/年改扩建项目进展顺利。重点工业产品产量稳中有增，生产原煤457万吨，原油13万吨，发电45亿度。完成工业增加值26.74亿元，增长21.4%。服务业结构优化。建成老龙潭·中华龙文化博览园和火石寨地质博物馆，须弥山石窟升级为国家4A级景区，固原市获得最美中国·特色魅力旅游目的地城市称号。与阿联酋皇家投资局达成建设六盘山旅游国际休闲度假区项目意向，对外开放迈出新步伐。西兰银综合物流园一期工程基本完成，新建大型商场2家、农家店和移民新村超市321家，商贸流通等传统服务业明显提升。完成三产增加值90.23亿元，增长12.5%。非公经济迅速发展。出台加快非公有制经济发展实施细则，拓宽融资渠道，支持改造升级，已占经济总量的66%。四是县域经济推进有效。各县(区)围绕资源优势和产业特色，抓典型，创亮点。原州冷凉蔬菜、马铃薯种薯产销两旺，工业园区建设和招商引资取得突破。西吉马铃薯、西芹实现产业化发展，月亮山生态建设效益可观。隆德中药材种植迅速扩规，“城中村”改造力度加大。泾源苗木培育和黄牛养殖品牌效益明显，休闲避暑旅游吸引力增强。彭阳红、茹河流域10万亩设施农业产业带规模喜人，农民收入增幅大。各县(区)初步形成了工农互补、城乡统筹发展的县域经济新格局。

　　二、城乡建设统筹推进，特色明显。城镇化率提高2.2个百分点，达到37.06%。一是城镇发展品质提高。首次召开推进城镇化工作会议，出台加快推进城镇化实施意见。政府投入9.1亿元，推进区域中心城市、县城和中心镇建设。发挥城市规划委员会作用，把好规划审查审批关，完成城市控制性详规和城市道路、供排水、供热、供气专项规划修编评审工作，强化规划的指导作用。拆除违法建筑1471户31.7万平方米，基本遏制了乱搭滥建行为。改造城市棚户区、老旧小区19万平方米。启动西南新区建设，征地1.2万亩，建设九龙路等道路16公里，九龙佳苑、市医院迁建等项目进展顺利。城市规划展示馆主体完工，火车站大桥和萧关路等市区道路建成通车。古雁公园一期工程顺利竣工，成为全区五市首座城市森林公园。宁南区域中心城市功能日趋完善，宜人景象正在形成。实施了西吉慈善大道、隆德星兴购物广场、泾源世纪购物广场、彭阳悦龙新区建设等一批大县城扩容提质工程。三营、兴隆、沙塘、六盘山和古城等中心镇建设特色更加明显。建设美丽村庄20个。开展环境综合整治和主干道路大整治大绿化，城乡面貌更加靓丽。二是水路电建设重大突破。宁夏中南部地区城乡饮水安全水源及连通配套工程加快建设，秦家沟水库建成使用，龙潭水库主体完工，中庄水库进展顺利，西吉应急供水工程建成通水，实现了群众40年的企盼和梦想。积极推进路网建设，东毛高速、西静、彭青一级公路和北环路建设进展顺利，固西高速公路及东环路、国道309过境段开工建设，新修农村公路1000公里。完成西南新区高压线路迁移，实施了城乡电网改造升级工程。三是生态建设扩面提质。全面推进水源涵养林种植、移民迁出区生态恢复治理、退耕还林补植补造、小流域综合治理，实施了长城梁二期和城乡大环境绿化工程，完成造林绿化43万亩，治理水土流失317.2平方公里，建成高标准绿色通道528公里。环境污染综合治理力度加大，出台农用残膜回收办法，回收率达40%;城市空气质量优良天数达92%，云雾山、火石寨保护区晋升为国家级自然保护区。

　　三、争取支持助推发展，成效突出。主动争取国家部委和自治区厅局政策、资金、项目支持，带动发展。一是政策争取有成绩。承办了六盘山片区扶贫攻坚部省协调推进会，提请国家部委解决的54个事项已有32项列入计划，市农业科技园区被科技部批准为国家农业科技园区。我市被列为自治区“三旧改造”试点城市，全市国省干线道路征地拆迁资金由地方承担一半改为自治区按标准补贴，极大减轻了地方财政支出压力。二是资金争取有成效。争取国家、自治区各类补助资金127亿元，有力支持了重点项目和民生工程实施。金融支持地方经济发展作用增强，年末存贷款余额分别达到271亿元和162亿元，存贷比达到59.8%，比去年提高4.3个百分点。三是项目争取有突破。多次到国家部委和自治区相关厅局汇报、争取项目，宁夏六盘山片区扶贫产业试验区建设被列入全国人大重点建议办理，提前开工城乡安全饮水配水项目和高速公路项目，公路投资占自治区年度公路投资的一半以上，破解发展瓶颈步伐加快。四是招商引资有成果。坚持市级领导带头招商，制定招商引资项目集中限时联办制度，成功举办和参加“宁夏固原·山东济南经贸合作推介会”、“福建百名企业家固原行”、“光彩事业六盘行”和中阿博览会等活动18次。引进实施项目226个，实际到位资金103.5亿元，增长33.9%。

　　四、民生改善继续推进，群众满意。认真落实自治区民生计划，15件民生实事全面完成。一是生态移民进度快见实效。严格落实“四不”要求，加快移民搬迁进程。县外搬迁移民9323户4.4万人，完成当年任务的117%。县内搬迁安置移民8063户3.5万人，累计配套设施种植17.8万亩、养殖圈棚8000座，提前两年完成“十二五”县内移民搬迁任务。二是扶贫攻坚措施硬见成效。编制了《六盘山集中连片特困地区(固原市)扶贫开发实施规划(2013—2017年)》，启动新一轮315个贫困村整村推进扶贫开发工作，整合5.88亿元，落实“四到”扶贫措施，减少贫困人口7.2万人。西吉县以危房改造为主的基础扶贫、泾源县整乡推进扶贫试点和彭阳县产业扶贫模式在全区总结推广。三是民生实事落实好见实惠。召开全市教育工作大会，出台办好人民满意教育实施意见，调动各方尊师重教。新建、改扩建校舍21.3万平方米，救助贫困生、营养改善计划等政策全面落实;全力实施中小学校长教师能力提升计划和骨干教师建设工程，高考中考再创历史佳绩。加强人才工作，表彰奖励了首届“六盘英才”。成功举办“唱响中国梦·建设新固原”系列活动，开展广场演出、送戏下乡646场次;隆德县被命名为“中国社火文化之乡”。率先在全区建成669个标准化村卫生室，与宁夏医科大学建立了培养医疗人才平台。上调医疗保险筹资标准，提高报销比例，启动城乡居民大病医疗保险试点。制定加强城乡低保管理服务意见;筹资1390万元，救助困难群众13.9万人。在全区率先试点国家计划生育特殊困难家庭帮扶项目，落实长效节育措施2.2万例，创建自治区“少生快富”工程整村推进村60个，实施“少生快富”工程3011例。转移就业30.57万人，创收37.09亿元。建设保障性住房5000套，重建受灾房2342户，改造危房危窑1万户，解决了近7万人的住房难问题。广泛开展食品药品安全示范创建活动，群众饮食用药安全得到保障。设立价格调节基金，控制物价上涨。城镇就业、水质达标、居民用气、公共交通等问题明显改善，生活质量提高。四是社会治理决心大见成效。“六五”普法扎实推进，“平安固原”创建活动深入有效，公众安全感满意度连续三年位居全区五市第一。下决心从源头治理安全隐患，筹资2813万元治理交通安全隐患41处，全年未发生较大安全事故，安全生产形势持续好转。面对20年不遇的严重雨涝灾害，及时启动防灾减灾预案，全力排查整治隐患，全市筹措3000多万元开展抗灾自救，妥善安置受灾群众，确保了城乡居民生产生活没有出大的问题。组织开展防震防火等应急救援演练，政府应对突发事件的能力增强。以“两创建、两争做”活动为载体，组织宗教人士外出考察、培训研讨，引导宗教与经济社会发展相适应，促进民族团结、社会稳定。顺利完成第九届村委会选举。军民共建、外事侨务、统计档案、气象人防、残疾人等事业取得新成效。

　　五、政府建设强基固本，廉洁务实。坚持立规矩、转作风、求实效，切实推进政府自身建设。一是重制度建设。修订并执行《市政府工作规则》《市政府常务会议规则》，健全工程建设招标、重大投资项目公示、专项资金管理、集体决策重大问题等制度，行政行为进一步规范。完善和健全听证制度，对市区天然气特许经营权进行了公开评定，反响良好。完善信访工作8项制度，畅通了群众诉求渠道。二是重作风建设。认真落实改进工作作风密切联系群众各项规定，政府文件减少20.5%，会议减少26.6%，临时议事协调机构由75个调整为10个，市本级“三公”经费下降20.4%。按规定清理办公用房，全面停建党政机关楼堂馆所。深入开展“三同六送六帮”活动，解决了基层群众的一些实际问题。扎实开展群众评议机关和干部作风活动。认真清理行政审批和收费项目，创优发展环境。三是重效能建设。整合4个园区成立经济技术开发区，理顺了工业园区、市区城管和园林管理工作机制。严格耕地保护制度，高质量完成国土部土地例行督察和卫片执法检查工作，土地管理工作更加规范。强化政府投资项目审计，节约资金7024万元。配合自治区完成债务审计，截止2013年底，全市政府债务余额59.5亿元，债务率34.8%，其中市本级债务余额20.6亿元，债务率44.7%;全市偿还政府债务16.6亿元，偿债率9.7%，其中市本级偿还5.7亿元，偿债率15%，总体保持在安全可控范围内。认真全面落实市委决定事项，办理市人大代表建议21件、市政协委员提案156件，办结率100%。推进政务公开，高质量办理网上政民互动信件3553件。四是重廉政建设。认真落实廉洁自律各项规定，廉政建设责任制深入推进。规范项目审批、资金安排和公共资源交易管理办法，接受社会监督。

　　各位代表，一年的经历和实践，让我们深切体会到：加快固原发展，必须坚持解放思想、更新观念;必须坚持实事求是、科学决策;必须坚持依法行政、和谐建设;必须坚持依靠人民、造福百姓;必须坚持团结奋进、誓拔穷根!一年的拼搏和收获，让我们真切感受到：做好政府工作，离不开自治区党委、政府和市委的正确领导、充分信任;离不开市人大常委会及代表、市政协及委员的有效监督、大力支持;离不开全市广大干部群众的积极参与、共同努力。在此，我代表市人民政府，向各位代表和委员，向全市各条战线上的劳动者、各民主党派、工商联、人民团体、社会各界和中央、自治区驻固单位、人民解放军、武警官兵及公安干警，向所有关心固原建设和发展的朋友们，表示崇高的敬意和衷心的感谢!

　　一年的工作既有发展和进步，也伴随着不少困难与问题：一是支撑经济快速增长的动力不足，产业层次低，规模以上企业少，缺乏打基础、利长远的大产业项目。二是城市建设中旧城改造项目建设成本高，征地拆迁难度大，投资发展环境有待优化。三是民间借贷不规范且风险加大，社会治理有待加强，影响社会治安的恶性案件易发、多发。四是民生改善需求大、财力弱，应对自然灾害的能力不强，扶贫攻坚任务艰巨。五是政府工作推进中还存在落实不到位，办事效率不高，行风评议靠后等。这些问题，是我们今后工作寻求突破的着力点。

　　各位代表，发展是永恒的主题，脱贫是坚守的目标，改革是持续的动力，进取是不变的精神。只要我们牢记重托、真情为民，就有力量源泉、精神支柱和开拓勇气，就能战胜发展中的一切艰难险阻!

　　2014年目标任务

　　今年是实现“十二五”规划目标的关键一年。做好各项工作，我市有以下重大机遇：一是发展机遇。党的十八届三中全会全面深化改革的总体部署，中央经济工作会议明确的稳中求进发展总基调，国家即将实施的“丝绸之路经济带”，加快宁夏内陆开放型经济试验区建设的战略决策，以及自治区党委十一届三次全会提出制定的宁夏空间发展战略规划，将拓展我市加快发展的空间。二是政策机遇。国家支持西部地区、老少边穷地区发展的政策力度继续加大;自治区出台深化改革推进经济社会发展若干问题的决定、产业转型升级和结构调整实施方案等政策，更加注重在扶贫攻坚、产业发展、城镇建设、危房改造等方面的投入，将支持我市加快发展。三是内生机遇。我市经过多年发展和积累，一些制约瓶颈逐步破解，形成的资源、生态、人文等优势蓄势待发，市第三次党代会确定的“把握一个主题、推进三个转变、实施六大战略”总体要求，以及配套出台的工业强市、城镇化发展、特色农业增效等意见和决定明确的发展思路、目标和工作重点已形成共识，全市上下务实为民、激情干事的氛围越来越浓，将助推我市加快发展。只要我们抢抓机遇、趁势而上，就一定能实现更高质量、更快速度、更好效益、更加惠民的发展。

　　今年工作的总体要求是：高举中国特色社会主义伟大旗帜，以邓小平理论、“三个代表”重要思想、科学发展观为指导，深入贯彻落实党的十八届三中全会、自治区党委十一届三次全会、自治区十一届人大三次会议精神，认真领会落实中央和自治区经济工作会议部署，按照市委三届四次全委会稳中求快总基调和“抓好‘六项重点’、破解‘三大难题’、推进‘五个创新’”总要求，以深化改革为动力，以强市富民为目标，以建设服务型政府为核心，以抓好100个重点项目和20件民生实事为重点，推进产业升级、城乡发展、项目建设、民生改善和改革开放五项工作，提升经济发展、城镇管理、工作创新、社会治理和政府服务水平，促进全市经济社会平稳健康发展，为实现建设新固原、与全区同步进入全面小康社会目标艰苦创业(简称五推进五提升)。

　　主要目标是：地区生产总值增长12%左右，地方公共财政预算收入增长15%(市本级增长15%)，全社会固定资产投资增长30%左右，城镇居民人均可支配收入增长12%，农民人均纯收入增长13%，社会消费品零售总额增长14%;城镇登记失业率控制在4%以内，居民消费价格涨幅控制在3.5%左右，人口自然增长率控制在10.5‰以内，完成自治区下达的节能减排任务，力争城市空气质量优良天数稳定在95%以上。各项目标的确定，既贯彻了国家和自治区经济发展稳中求进的总基调，体现了经济增长要有质量和效益，考虑了农村改革红利扩大、促进农民增收的因素，又兼顾到经济下行压力继续存在、推动经济快速增长的新动力有限。确定GDP增长12%左右，主要基于我市基数低，实现与全区同步进入全面小康社会的任务重、差距大，经济增速理应高于全国增长7.5%左右、全区增长10%左右。实现目标任务，重点抓好以下工作：

　　一、推进产业升级，提升经济发展水平

　　产业升级是我们拔除穷根的根本举措，制定并实施我市《产业结构调整和优化升级方案》，指导今后产业健康发展。安排6000万元推动产业优化布局、扩大规模、提质增效。

　　助推特色农业增效。以建设国家农业科技园区为抓手，安排2000万元，按照“一县一业”和“产加销”一体化模式，发展草畜、马铃薯、冷凉蔬菜、中药材和特色农产品加工五大产业集群。其中安排1000万元，扶持肉牛养殖，新增肉牛10万头，达到100万头以上。优质马铃薯稳定在100万亩以上。每个县(区)建设一定规模的永久性蔬菜基地，新增设施及大田蔬菜2.5万亩，达到55万亩。大田中药材达到13万亩。苗木达到36万亩。支持发展原州天启薯业、西吉华林和佳立公司等区、市农业产业化龙头企业15家，培育农民专业合作社50家，引进5000万元以上特色农业项目5个，农产品加工转化率力争达到50%。每个县(区)建设1个农业机械化示范园区，农业机械化率增长2个百分点。农业增加值增长5%以上。

　　助推工业经济扩容。全力推进工业强市战略，安排3000万元，加快园区发展和项目建设，重点发展能源、节能环保新材料、农产品加工、战略性新型产业四大主导产业集群。完成盐化工示范区、轻工产业园和五县(区)产业园基础设施续建工程，探索建立与发达地区合作共建园区机制，新签约投资过亿元项目10个。开工建设中铝煤电铝一体化、福宁广业马铃薯淀粉深加工、家道公司全谷物食品产业园、申科电力设备制造等项目，加快建设王洼煤矿千万吨级矿区、欣正天然气液化、原州邦德电动自行车二期、西吉风光互补LED灯具、隆德佐源集团菊芋深加工等项目，建成金昱元20万吨PVC、恒辉实业轻工建材市场、银南建筑装备制造电梯及自动旋转门、晶得10兆瓦光伏电站等项目。扶持中小微企业改造升级，延长产业链，提高附加值。工业增加值增长20%，其中规模以上工业增加值增长16%。

　　助推服务业提升。安排文化旅游发展资金1000万元，完善基础设施，提高服务质量，全力打造内陆开放型经济试验区的“旅游特区”和“国家级旅游扶贫示范区”。完成六盘山红军长征景区游步道景观工程和重点景区基础设施建设工程，尽早开工建设六盘山旅游国际休闲度假区，根据条件适时开工建设博物馆文化产业城一期工程。争取建设福银高速李庄至泾河源镇旅游专线，争取改扩建须弥山—火石寨—六盘山和固原—中卫、固原—平凉旅游通道，构建六盘山旅游圈。扩大对外宣传，力争接待游客250万人次，实现旅游社会总收入12亿元。完成西兰银综合物流园二期工程，打造特色商业街区6个，建成西南新区标准化菜市场，改造提升商贸餐饮等传统服务业。鼓励兴办金融机构，创新金融发展，支持产业升级和农民增收。积极发展电子商务，推进特色农产品网上交易，扩大六盘山农产品市场占有率和知名度。三产增加值增长11%。

　　助推非公经济壮大。认真落实加快非公有制经济发展实施细则，优化服务环境。积极争取国家项目，扶持做大做强一批骨干民营企业。改革工商登记制度，拓宽非公经济发展领域，激发发展活力。力争非公经济增长15%。

　　助推县域经济发展。继续落实市级领导包抓县(区)和重点工作责任制，支持各县(区)发挥优势，整合资源，突出特色，在现代农业、城镇建设、环境改善、危房改造等方面打造精品工程、亮点工作，壮大综合实力。继续抓好县域经济观摩、督查考核，形成优势互补、竞相发展的良好态势，提升县域经济发展水平。

　　二、推进城乡发展，提升城镇管理水平

　　落实中央城镇化工作会议精神，按照全市加快推进城镇化实施意见，突出城乡统筹、产城融合发展，构建“1411”城镇体系，全力打造“红色六盘、绿色固原、避暑胜地”城市形象。

　　加快城乡规划修编和管理。根据《宁夏空间发展战略规划》，统筹规划生活居住、产业布局、生态建设空间布局，完善城市总规，编制相关专项规划，修编控制性详规，组织编制乡镇、美丽村庄建设规划。各县(区)成立城乡规划委员会，把好规划关。制定城镇、乡村规划和建设管理办法，依法加强城乡规划和管理，切实维护规划的权威性和严肃性。

　　加快区域中心城市建设。按照生态园林和文化旅游城市的特色定位，完善城市功能。开工建设福银高速固原南出口、古雁路下穿福银高速公路桥，建成东环路、北环路、固胡路。加快西南新区建设，完成九龙佳苑、市医院迁建等项目主体工程。改造老城棚户区1万户。推进“四大主题公园、四条生态山脉、四大水库湿地”建设，开工建设古雁公园二期、儿童公园、九龙公园和北海湿地公园，提高宁南区域中心城市综合承载能力。

　　加快城镇和美丽乡村建设。合理配置资源，推动县城、中心镇和美丽村庄扩容提质、协调发展。重点实施西吉文化广场、隆德灵龟山公园一期、泾源龙脊山森林公园、彭阳悦龙山新区等项目。年内集中打造黄铎堡、将台、联财、泾河源、王洼等中心镇和25个美丽村庄。改革城乡户籍管理制度，完善社保、医保、住房保障政策，促进农业转移人口就近市民化。

　　加快城市精细化管理工程建设。进一步改革理顺城市管理体制机制，推进市区共建共管。组建城市综合执法机构，提高城市管理效率和水平。以建设智慧固原为目标，开展数字城管工程建设。全面启动国家园林城市、环保模范城市、水生态文明城市、文明城市和自治区卫生城市“五城”联创活动，不断提升城市品质。

　　三、推进项目建设，提升工作创新水平

　　项目建设是破除固原发展瓶颈，实现强市富民、跨越发展目标的根本手段，必须紧抓不放，常抓不懈。

　　抓好前期工作。抓好东线高速原州高台至彭阳高寨段、泾河源至华亭高速、彭阳至麻子沟圈(庆阳界)公路项目和宝中铁路复线建设项目的前期工作。组织开展“准备之冬、开工之春”活动。重点抓好项目开工前的征地拆迁、土地报批、规划设计和招投标工作，争取上半年全部开工建设。启动“十三五”规划编制前期工作，论证储备一批增后劲、利长远的大项目、好项目，争取更多项目进入国家和自治区的大盘子。

　　抓好项目建设。以提高项目建设进度、质量、服务为抓手，实施100个重点项目(续建44个，新建56个;具体项目见附件1)。着力破解水、路、绿“三大难题”，提升可持续发展保障能力。围绕建设“智能水网”，加快建设中南部城乡饮水安全水源及连通配套工程、清水河干流防洪工程(固原段)、中型水库抗旱水源提升改造工程、20座病险水库除险加固和10处农村饮水安全工程，完成盐化工示范区引水工程，全力保障生活、生产、生态用水安全，保持水价合理。围绕建设“致富路网”，加快建设东毛、固西高速和彭青、西静一级公路等项目，完成国省干道309过境段改造工程，新建农村公路500公里，打造高效便捷、互联互通的高速交通网络。围绕建设“生态林网”，实施长城梁绿化续建、饮马河流域生态治理及中庄水源地保护、西吉月亮山水源涵养生态修复项目;每个县区争取修建一座休闲生态公园;把植树造林作为生态建设的“一号任务”，鼓励发展林药、林草经济，完成造林绿化、封山育林和移民迁出区生态恢复治理100万亩;争取国家水资源和生态补偿试点，探索建立矿产资源补偿机制，推进大六盘生态长廊建设，全力保护好山青水秀、地绿天蓝的“绿色固原”形象。

　　抓好资金保障。争取国家、自治区补助项目资金70亿元，争取融资10亿元，其中发行债券7亿元。支持鼓励社会资金参与项目建设，争取金融机构存贷比达到75%。创新招商机制，完善优惠政策，实行招商引资项目全程代办服务，引进一批技术含量高、产品附加值高、财税贡献率高、劳动就业率高的项目，力争招商引资实际到位资金达到130亿元以上。

　　抓好协调服务。落实重大项目推进工作办法，建立项目争取、建设、管理、协调服务联动机制。落实市级领导、部门领导包抓重点项目责任制，对重大项目建设一线指挥，现场协调解决问题，营造良好的项目建设环境。

　　四、推进民生改善，提升社会治理水平

　　牢固树立民生改善投入只增不减、市民生活质量只提不降、民生实事覆盖面只扩不缩的为民理念，竭力办好富民、便民、惠民、安民的20件实事(具体内容见附件2)。

　　加大生态移民致富力度。加强协调对接，完成县外移民25095人，争取全面完成“十二五”生态移民任务。县内移民抓好基础配套、产业培育、社会治理和生产生活方式转变，实现管得好、逐步能致富目标。

　　加大扶贫开发攻坚力度。抓好六盘山片区扶贫攻坚部省协调推进会政策和项目的对接落实。开展河川、红耀、杨河、六盘山、红河5个乡(镇)整乡推进扶贫试点。加强资金整合和闽宁协作、社会帮扶工作，整村推进扶贫开发125个贫困村，减少贫困人口7万人以上。严控政策外生育，实施“少生快富” 工程3000例，创建“少生快富” 示范户500户。

　　加大危房危窑改造力度。编制全市危房危窑改造实施方案，把危房危窑改造与推进整村扶贫开发、建设美丽乡村结合起来，按照县区负责、统一规划、统一标准、分类安置、就地翻建、因地制宜的原则，完成危房危窑改造30000户，切实改善群众居住条件。

　　加大尊师重教力度。认真落实办好人民满意教育实施意见，启动新一轮学前教育三年行动计划，迁建市幼儿园，新建6所县城幼儿园，每个乡镇办好1所中心幼儿园。加快标准化学校建设，争取立项新建西南新区中学，扩建固原二中、回中、特教中心，实施隆德四中、西吉二中等初中校舍和薄弱学校改造项目，迁建彭阳职中。争取立项建设六盘山高等职业技术学院。推进民办教育、特殊教育发展。加强教师队伍建设，提高教育质量。

　　加大社会治理创新力度。创新有效预防和化解社会矛盾体制机制，依法化解涉法涉诉信访案件。严厉打击违法犯罪，增强群众安全感。依法加强社会组织管理，加强民间借贷监管，规范市场经济秩序。建成市应急指挥中心，健全防灾减灾救灾平台。开展安全生产重点领域、突出问题大整治，有效遏制重特大事故发生。强化食品药品全程监管，保证群众舌尖上的安全。牢牢把握共同团结奋斗、共同繁荣发展(简称“两个共同”)主题，树立汉族离不开少数民族，少数民族离不开汉族，各少数民族之间也相互离不开(简称“三个离不开”)的思想，依法依规加强宗教事务和活动管理，促进民族团结、宗教和顺。重视国防和民兵预备役建设。支持中央、自治区驻固各单位和人民团体为全市经济社会发展做出新贡献。

　　五、推进改革开放，提升政府服务水平

　　改革开放是我市跨越发展的强大动力，也是用好机遇、破解难题的关键一招。

　　改革开放促发展。认真落实市委三届四次全委会确定的推进“五个创新”部署，坚持解放思想、改革开放、创新驱动，积极稳妥推进以经济体制改革为重点的各项改革，营造公平、开放、有序的市场环境，使市场在资源配置中起决定性作用和更好发挥政府作用。完成农村土地承包经营确权颁证，加快农村土地流转，盘活农村经济发展。改革完善城乡一体发展机制、土地征用政策和目标管理考核办法，建立科学有效的激励机制。抢抓“丝绸之路经济带”和“两区”建设机遇，提升固原扶贫开发示范区承载力。承办陕甘宁蒙毗邻地区联席会议，加强区域交流合作，扩大对内对外开放。争取自治区召开第五次固原工作会议，出台推进改革创新，加快固原发展的政策机制。

　　转变职能强服务。加强向先进学习、向市场放权、向民生聚力，促进行政理念由管理向服务转变。按照统一精简效能原则，转变政府职能，深化政府机构改革，优化机构设置、职能配置和人员调整，理顺权责关系，提高综合治理能力。实现机构、人员、行政经费只减不增，降低行政成本。按照政事分开、管办分离的原则，统筹推进事业单位分类改革，建立事业单位法人治理结构。今年2月底前提出全市政府机构改革方案，上半年完成改革任务。

　　简政放权优环境。以处理好市场与政府的关系为核心，以打造比东部更优、西部最优发展环境为目标，深入推进行政审批制度改革。建立政府权力清单和负面清单制度，负面清单以外的经济活动一律取消审批，凡直接面向基层的经济社会事项一律下放县级政府，凡市县内的企业核准项目一律实行备案制。清理行政审批事项和行政事业性收费项目，实现“三个减少60%以上”，即审批事项减少60%以上、收费项目减少60%以上、审批事项和民生服务事项办理时限减少60%以上。保留的事项和民生服务事项全部进驻政务服务中心和民生服务中心，实行一门受理、并联审批、多证联办，逐步推行网上审批办理，提高审批和办事时效，打造宁夏用时最短、办事最快的政务环境。

　　改进作风树形象。进一步完善调查研究、专家咨询和市民听证等办法，严格执行《市县(区)党委重大事项集体决策制度(试行)》，提高民主决策水平。坚持依法行政和民主议政，自觉接受人大、政协和社会各界监督，积极推进政协协商工作计划落实。严格落实中央、自治区和市委改进作风各项规定，进一步改进会风文风，会议和文件数量再压缩20%以上。厉行节约，反对浪费，实现市级 “三公经费”压减10%以上。按照自治区党委和市委的统一部署，扎实开展党的群众路线教育实践活动，认真解决“四风”方面存在的突出问题。重视和加强政府系统党建工作，充分发挥党员干部的带头示范作用。倡导艰苦创业、务实苦干作风，深入基层、深入一线，帮助群众解决实际困难。加强责任、诚信政府建设，牢固树立责任意识，勇于负责，敢于担当。做到守土有责，承诺必兑，偿还旧账，控制新债，多留财富，不留包袱。加大明察暗访和行政问责力度，严惩不作为、乱作为行为，严肃查处各类损害群众切身利益的违法违纪案件。严格执行廉政建设责任制，强化审计监督，提高资金使用效率;落实政府类投资项目招投标制度，坚持用制度管权管事管人;扩大政务公开范围，实现决策、管理、服务、结果 “四公开”，从源头上预防和遏制腐败。深入推进群众民主评议行风政风活动，适时开展电视问政、微博问政，接受市民和企业评价，引导督促政府工作人员履职尽责、用心想事、激情干事、竭力成事。树立政府科学执政、依法行政、务实勤政、廉洁从政的良好形象。

　　各位代表，改革号角催人奋进，脱贫任务时不我待。让我们在自治区党委、政府和市委的坚强领导下，高举中国特色社会主义伟大旗帜，深入实践科学发展观，坚定信心，艰苦奋斗，为实现建设固原、与全区同步进入全面小康社会目标而努力奋斗!
